# A pilot study in intraparenchymal therapy delivery in the prostate: a comparison of delivery with a porous needle vs standard needle

**DOI:** 10.1186/s12894-018-0378-8

**Published:** 2018-07-28

**Authors:** Martin L. Brady, King Scott Coffield, Thomas J. Kuehl, Raghu Raghavan, V. O. Speights, Belur Patel, Scott Wilson, Mike Wilson, Rick M. Odland

**Affiliations:** 1 Therataxis, LLC, Baltimore, MD USA; 2 Department of Surgery, Division of Urology, Scott & White Medical Center, Temple, TX USA; 3 Department of Obstetrics & Gynecology, Scott & White Medical Center, Temple, TX USA; 4 Department of Pathology, Scott & White Medical Center, Temple, TX USA; 5 Texas A&M Health Science Center College of Medicine, Temple, TX USA; 6 Departments of Obstetrics & Gynecology, Pediatrics, and Molecular & Cellular Medicine, Texas A&M Health Science Center College of Medicine, Temple, TX USA; 7 Twin Star TDS, LLC, Lexington, KY USA; 8 Department of Otolaryngology, Hennepin County Medical Center, University of Minnesota, Minneapolis, MN USA

**Keywords:** Prostate, Porous Needle, Infusion, Imaging, Distribution

## Abstract

**Background:**

New biologic therapies directly injected into the prostate are in clinical trials for prostatic diseases. There is a need to understand distribution of injected therapies as a function of prostatic anatomy, physiology, and device design.

**Methods:**

A needle with a porous length of customizable-length was tested and its performance compared with a standard needle. Injections of magnetic resonance contrast reagent were placed into ex-vivo human prostates after surgical excision in standard of care therapy for invasive bladder cancer patients. Magnetic resonance images were acquired using sequences to quantify volume delivered, distributed, and backflow.

**Results:**

Magnetic resonance images analysis revealed heterogeneity distribution with injection into the specimens. There was low resistance to flow along ductal pathways and high resistance to flow into glandular nodules and smooth muscle/fibrous parenchyma. Data confirm previous studies showing injection loss via urethra backflow, urethra, and prostatic ducts. Tissue fraction of dose was significantly higher with porous needle compared with standard needle (*p* = .03). We found that a greater volume of distribution divided by the amount infused (Vd/Vi) increased by 80% with the porous needle, though no statistically significant association due to small sample size.

**Conclusions:**

This study demonstrated that prostatic tissue is anatomically heterogenic and limits distribution of needle injection. There is greater distribution in the ex-vivo prostate using a porous needle. The complexity of intra prostatic flow pathways suggests preoperative imaging and pre-treatment planning will enhance therapy**.**

**Electronic supplementary material:**

The online version of this article (10.1186/s12894-018-0378-8) contains supplementary material, which is available to authorized users.

## Background

Benign prostatic hyperplasia (BPH) and prostate cancer are two major diseases in men [1, 2]. Symptomatic BPH occurs in two-thirds of men by age 80. One in seven men has prostate cancer diagnosed; it is a common cause of cancer death in men later in life. Though low risk prostate cancer is more common, intermediate and high risk prostatic cancer remain morbidity and mortality risks for one in nine men between 50 and 80 years of age. Radical prostatectomy and external or implant radiation therapy (standard of care) are associated with serious side effects upon a man’s quality of life. Common surgical and medical BPH therapies may fail or cause similar side effects. There is ample evidence of need for improved outcomes in both diseases.

Prostatic injection of biologicals to treat BPH and prostate cancer is a potential pathway to reduce serious side-effects of commonly used therapies. A major challenge for developing therapies based on prostatic injection is lack of predictable control of distribution of the injected agent into a specialized muscular organ that may have anatomic alteration of the prostatic ductal continuity, invasion and distortion of prostatic fibromuscular tissue, and ducts with intermediate and high risk prostatic adenocarcinoma. Prostatic adenoma enlargement also alters the glandular ductal drainage and prostatic fibromuscular tissue with compression and fibrous barriers rather than invasion. However, the impetus to develop minimally invasive therapies for benign and malignant disease has grown with recent advances in therapeutic related technologies of imaging, molecular innovation, and the interest of limiting the side effects of therapy for active and engaged patients. Intraprostatic injections have recently been explored, as these can be performed under local anesthesia. There has also been interest in development of two agents, Fexapotide triflutate (NX-1207) for symptomatic BPH and topsalysin (PRX-302) for BPH and organ confined prostatic cancer. Both have shown good safety profiles and early efficacy in phase II studies [3].

The goal of this study is to describe and define the distribution of liquid agents injected in ex-vivo prostates harvested at radical cystectomy. We aim to highlight a preliminary comparison of two different devices for infusion of fluid into prostate.

## Methods

### Devices and equipment

**Fig. 1:**
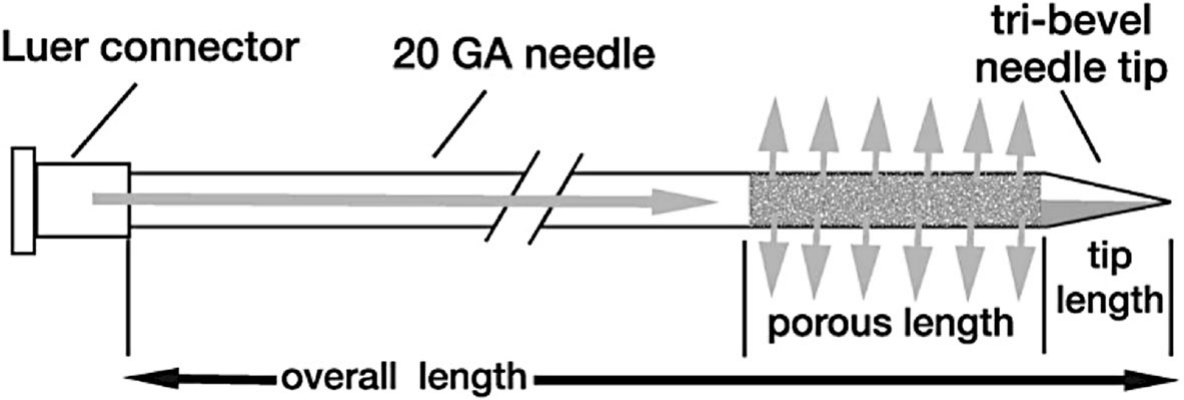
Includes an appearance of a standard needle though the porous segment is readily visible with variable length. Study needle was 20 Ga with a tri-bevel tip for penetration ease and single lumen with standard luer-lok connector

Institutional review board approval was obtained to undertake the tissue harvesting and injection of the prostates in this study. Inclusion criteria included 18 years of age, prior diagnosis of invasive bladder cancer, lack of prior bladder radiation, prostate radiation, bladder chemotherapy, prostate chemotherapy or prostate surgery, and the granting of IRB-approved tissue harvesting consent from each patient. We used a standard, control needle and a porous, investigation needle (Fig. 1) in this study, both 20 gauge MP35N alloy with a length of 20cm and plastic luer fitting on proximal end. The control needle was end port design with single bevel at the distal end. The distal end of the porous, investigational needle has a solid tri-bevel tip. The porous length starts 3mm proximal to the tri-bevel tip point and was 1–2 cm long in this study. The porous length is a thermomechanical formed metallic structure designed to provide distribution along the entire porous length.

### Sample preparation; device insertion and placement

Human prostates were harvested at radical cystoprostatectomy from 16 consented patients with muscle invasive transitional carcinoma. After bladder and seminal vesicles removal, the prostates were cooled in ice slush and transported to the research laboratory for processing. Prostate volumes were estimated using measurements of thickness, depth, and width, using the formula for an ellipsoid. Each prostate was positioned in a magnetic resonance (MR) compatible container encased in insulating foam, stabilized with the apex perpendicular to a brachytherapy guide template. The urethra was filled with insulating foam to permit localization without occlusion. The stabilized prostate was placed inside a body coil using a 3T Siemens Trios (Siemens, Enlagen, Germany) for imaging. High-resolution, serial axial sections were obtained with 1mm thick slices to plan the positioning of two devices for infusion. The pulse parameters for the FLASH acquisition were TR = 30 ms, and averaging acquisitions with TE from 2.2 to 23.2 msec. The voxels were isotropic and the resolution was 0.95 mm. Needle placement was performed by a single urological team (KSC, TJK) experienced with image guided prostate needle placement. The infusions targeted the mid transition zone of the prostate, since that is where it is easiest to envisage placing a needle traversing the peripheral and transition prostatic zones in a bilaterally symmetric manner. The insertion depth of the porous needle and standard needle was the same, about 20 mm, which was typically a few mm less than the dimension of the prostate along the trajectory of the catheter in the urethra. Each device was used to infuse from 1—1.5mL of modified Galbumin™ (gadolinium-labeled albumin; Biopal, Inc., Worcester, MA) (Additional files 1 and 2), using a duel channel syringe pump (Model KDS LEGATO 210, KD Scientific, Holliston, MA) during a 10-minute interval in all but four of the prostate specimens and during a 100-120 minute infusion in four to allow a more detailed evaluation of the distribution patterns. The infusion technique and times were selected to control pressure delivery into the prostates. The injected molecular agent was used to simulate molecular size of potential therapeutic agents. Serial dynamic images were obtained to document distribution through the gland in relation to anatomic landmarks and lesions. After each infusion, additional high-resolution images were obtained to determine quantitative distribution maps of infused contrast agent. The prostate was then removed from the container and immersed in formalin fixation. The prostate and urethra were processed for anatomic evaluation and sections were photographed and blocked for histologic examination.

### Contrast agent infusions and imaging

Galbumin™ served as a surrogate for the proteins used in therapies for prostatic disease. This reagent was infused at a concentration of 25% of the supplied Galbumin™, diluted 1:3 with saline solution. The resulting concentration was 0.0845mmol/L. The infusate was prepared in a 10mL volumetric flask by adding full volume from two vials with 25mg/mL of Galbumin™ in each vial, along with 100μL of food coloring and filling with phosphate buffered saline. Four separate vials using Glowing Galbumin™-Fluorescein infusate from Biopal, Inc. were prepared and placed in small tubes located within the field of view of the prostate. The composition of these vials varied from 0—2.5mg/mL of Gd-conjugated albumin. The four vials, each holding about 300μL of the markers, were placed below the prostate in the container used in the MR scanner. The prostates were cooled and imaged with contrast injection within 6-8 hours from harvest. Imaging with sequencing was acquired before infusion and once after the infusion at either 10 minutes or 100-120 minutes (in four prostates) after initiation of infusion using T1 maps computed from pairs of 3D FLASH MR scans at flip angles of 6 and 34° using the variable nutation method [4, 5]. B1 field inhomogeneities were corrected using a method for measuring said angles [6]. We measured the concentration of MR reagents by the method described by Brady et al for in-vivo infusions [7]. Gadolinium concentration C was computed from the T1 maps using the equation 1/T1 = 1/T10 + R1C, where relaxivity is denoted R1.

### Line pressure measurements

The line pressures were measured using transducers placed between the infusion pump and 25ft of high pressure tubing. The tubing allows for instruments to be located outside of the 25 gauss line of the MR unit.

### Data analysis

The volume of distribution V_d_ was estimated as the volume of the voxels containing a measureable concentration of gadolinium tracer and was used to compute the ratio V_d_/V_i_, where V_i_ is the volume of fluid infused. In calculating V_d_, we applied a threshold to the concentration instead of applying a threshold to the T1-enhanced image of the contrast reagent. For Galbumin*™*, the threshold was 0.002mmol/L, or 4.8% of the infusate concentration (this was the minimum computable concentration). In the case of CellTrack*™*, the threshold was 0.8% of the infusate concentration. Thus, V_d_ is the total volume of all voxels that contain at least this concentration of reagent. Increasing or decreasing this threshold tended to decrease or increase V_d_, respectively.

Comparison of the fraction of contrast delivered to prostate tissue with each needle type was tested with a two sided T test, corresponding to the conservative assumption that there is no a priori reason to favor the porous device delivering more or less infusate than the standard needle into a specialized muscular organ that was similar on both sides of injection.

Comparison of the ratio of volume of distribution to volume infused with each needle type was tested with two-sided T test, corresponding to the conservative assumption that there is no a priori reason to favor the porous device delivering more or less infusate than the standard needle into a specialized muscular organ that was similar on both sides of injection.

## Results

### Histopathological results

The histopathological analysis of each prostate was performed by a single pathologist utilizing the laboratory standard for prostate surgical specimens submitted with cystectomy for invasive transitional cell carcinoma. Final pathological reports revealed no incidental prostate cancer in the prostate specimens. There was a single transitional cell carcinoma focally extending into the proximal prostatic urethra without invasion into the prostatic stroma.

### Backflow

**Fig 2:**
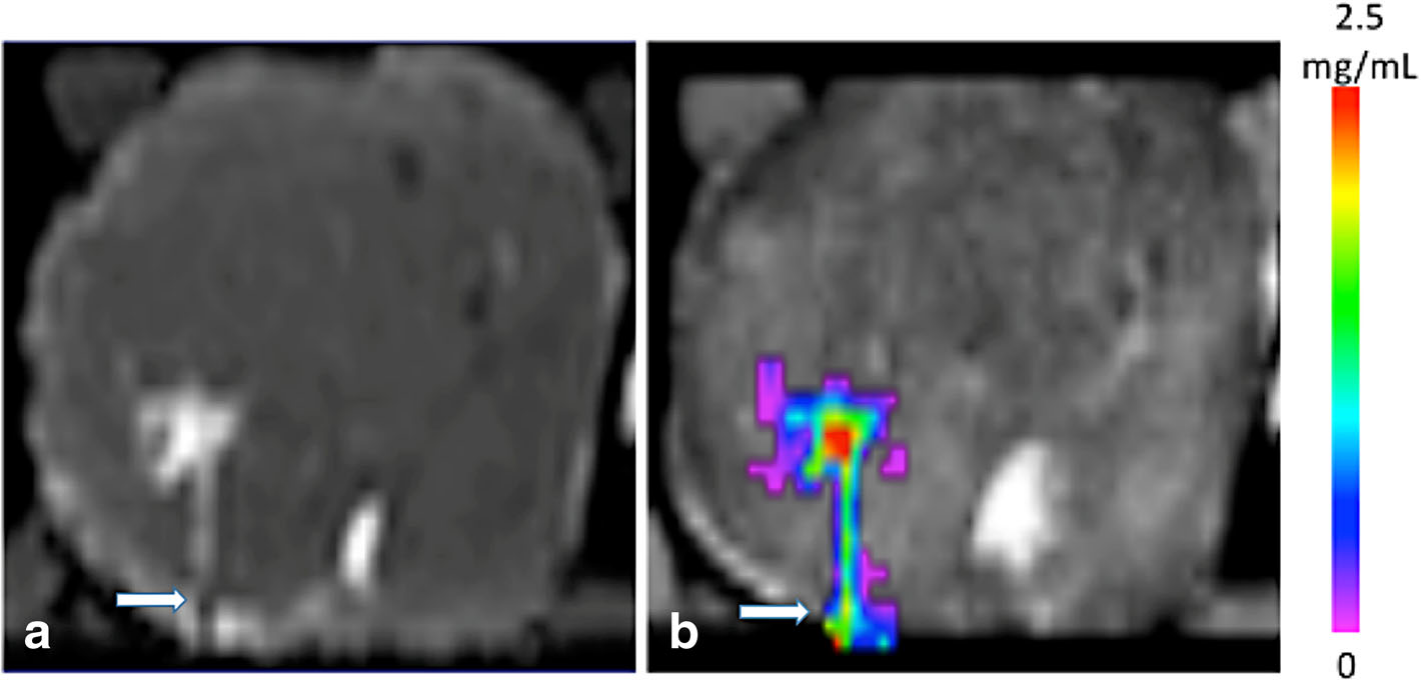
Image depicts standard needle infusate backflow (**a**) along the needle sides with concentration distribution (**b**) seen at highest in red and lowest in lavender. The Gadolinium distribution is seen in Fig. **a**. The concentration distribution is seen in Fig. **b**

Backflow is a well-known phenomenon due to the interaction of tissue elasticity with fluid flow [8]. However, there was essentially no measureable backflow beyond the proximal extent of the porous segment when the porous needle was used (Additional files 1 and 2). However, the standard needle did exhibit backflow (Fig. 2). Sometimes, there are other preferred pathways that prevent backflow reaching the prostatic capsule. Nevertheless, this remains a concern for needle infusions particularly at higher flow rates.

### Outflow

**Fig. 3:**
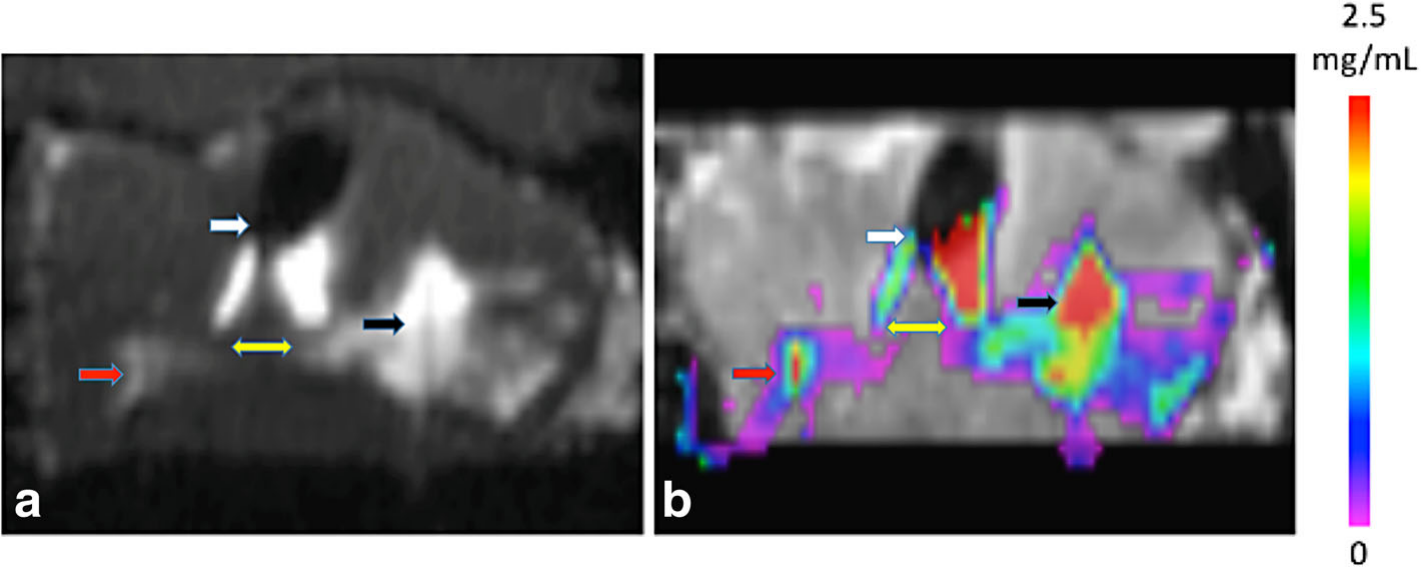
Left (**a**): T1 Gadolinium injection image with standard needle (red arrow) and porous needle (black arrow) pointing to the linear needle image entering the prostate from lower edge of prostate. White arrow depicts infusate movement to the urethra. Yellow arrow depicts channels of infusate from the injection site to the urethra. Right (**b**): Gadolinium concentration map showing outflow moved from the injection site to urethra (dark oval upper center) and subcapsular region in both figures. Note larger porous needle infusate distribution and concentration (red) on right compared to standard needle on left

We use the word outflow in a very restricted sense to mean volume transmission of particle (tracer in this case) beyond the prostatic capsule or into the urethra before the prostate or the target zone (e.g. the peripheral zone of the prostate) is filled with infusate (Fig. 3). With little spread within the parenchyma, much of such outflow can be avoided if backflow is well contained. However, as shown in Tables 1 and 2, only a small fraction of the total infused amount of tracer stays within the prostate.

**Table 1:** Comparison of fraction of contrast delivered to prostate tissue

Device vs needle	Fraction in tissue (mean with SE)	N	*p*-value
Device 1cm length of porous segment	0.27 ± 0.12 (0.24 ± 0.14)	7 (8)	0.013 (0.05)
Device 2 cm length of porous segment	0.33 ± 0.24	5	0.035
Needle	0.10 ± 0.11	8	0.010 (0.02)

The numbers in parentheses use an infusion not included in calculating the other entry in the same element of the table (see text for further explanation). The *p*-values are two-sided, corresponding to the conservative assumption that there is no a priori reason to favor the porous device. The entry for the *p*-values compares the device in the row to the standard needle: the third row is a comparison where both porous devices are aggregated in the comparison

**Table 2:** Comparison of ratio of Volume of distribution to Volume infused

Device vs needle	Vd/Vi	N	*p*-value
Device 1cm length of porous segment	1.32 ± 0.67 (1.17 ± 0.74)	7 (8)	0.211 (0.365)
Device 2 cm length of porous segment	1.46 ± 0.84	5	0.194
Needle	0.81 ± 0.81	7	0.116 (0.192)

The numbers in parentheses use an infusion not included in calculating the other entry in the same element of the table (see text for further explanation). The *p*-values are two-sided, corresponding to the conservative assumption that there is no a priori reason to favor the porous device. The entry for the *p*-values compares the device in the row to the standard needle: the third row is a comparison where both porous devices are aggregated in the comparison

### Preferred pathways

**Fig. 4:**
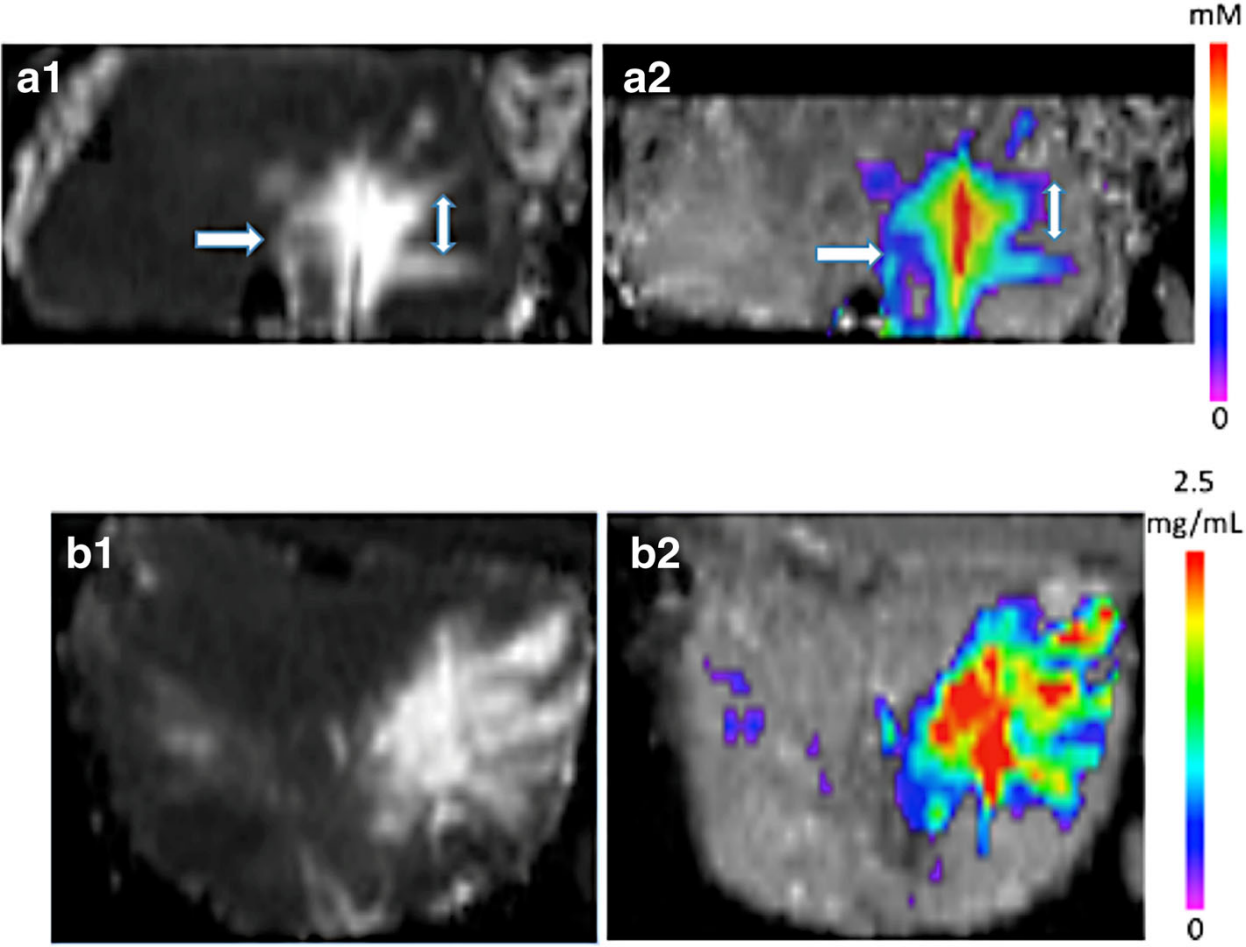
Top (**a**) demonstrates T1 Gadolinium injection image with porous needle on left showing distribution of contrast along ductal or directional pathways in the prostate leading to infusate loss to the outside as well as into the urethra (lower mid image). Right image demonstrates Gadolinium concentration map with contrast concentration along ductal or directional pathways in the prostate with less high (red) concentration in center of infusate due to loss to urethra and subcapsular. Bottom (**b**) demonstrates T1 Gadolinium injection image with porous needle showing greater distribution on left and greater concentration (note red in center imageL) on the right with less ductal or directional preferred outflow loss of contrast.

The ductal or directional pathways in the prostate seem also to lead to infusate loss to the outside as well as into the urethra (Fig. 4). The aim of a good delivery system is to reduce infusate loss and effective distribution to the targeted area.

### Obstacles

**Fig. 5:**
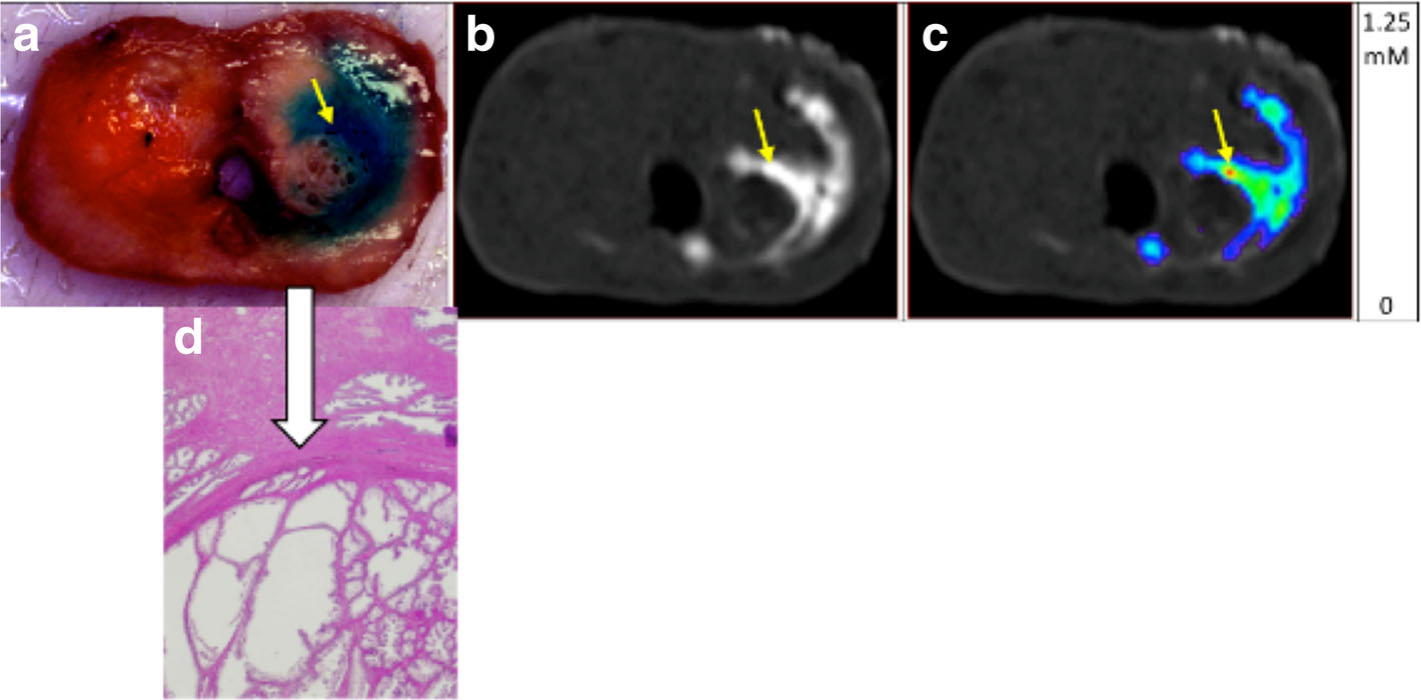
Top (**a**) demonstrates T1 Gadolinium injection image with porous needle on left showing distribution of contrast along ductal or directional pathways in the prostate leading to infusate loss to the outside as well as into the urethra (lower mid image). Right image demonstrates Gadolinium concentration map with contrast concentration along ductal or directional pathways in the prostate with less high (red) concentration in center of infusate due to loss to urethra and subcapsular. Bottom (**b**) demonstrates T1 Gadolinium injection image with porous needle showing greater distribution on left and greater concentration (note red in center image L) on the right with less ductal or directional preferred outflow loss of contrast

Unlike preferred pathways, obstacles such as glandular or stromal nodules appear either highly resistant to fluid flow throughout their volume or are surrounded by a fibrous barrier preventing ingress or efflux. Figure 5 displays post-infusion images showing (top images from left to right) infused dye, T1-weighted MRI, and computed concentration in a single axial section. This figure shows a clear avoidance of a region in the prostate: it is likely enclosed by a relatively impermeable fibrous and/or muscle tissue barrier. In the few cases where the needle or porous catheter is inserted into such a nodule, flow within the nodule is limited. This may also be due to the nodule itself being of high resistance. We cannot sharply distinguish between these possibilities from the imaging data; though in T2 imaging (which would show water as high intensity), there tends to be a thin dark band surrounding the nodule which would favor the hypothesis of an impermeable tissue barrier around the region.

Figure 5 also show excellent correspondence between the MR distribution of the contrast reagent and the visible distribution of the vegetable dye. In addition, a microscopic image of the portion of the tissue at the border of the nodule is shown at the bottom. An indication of why the infusion failed to penetrate is the fibromuscular capsule that surrounds the cystic nodule (as designated by the arrow).

### Overall results

We summarize the simplest metrics from the infusions in Tables 1 and 2. These are the amount of tracer actually detected within the prostatic tissue compared with the total amount infused in Table 1 and the volume of distribution V_d_ calculated as discussed above relative to volume of infusion V_i_ in Table 2. The data presented in Additional files 1 and 2 were omitted in the computation of the statistics: (i) the infusions with Prohance™ which were done as preliminary experiments to refine the protocol; (ii) bolus infusions with the needle which were not continuous infusions; and (iii) one placement with the 2-cm porous needle which inadvertently missed the prostate entirely. Further, there was (iv) another placement with the 1cm porous device from which no infusate could be detected over the entire porous section. What we have done is to report the statistics separately with this one infusion witheld and then included.

We note that the distribution volumes are computed not by a threshold on the brightness of a contrast-enhanced image, as is customary, but on the computed concentration of the contrast agent itself (Table 2). Thus, they are somewhat more objectively measured than in the usual way, though varying this threshold will change the distribution volumes. However, the dose is not dependent on any arbitrary threshold (Table 1). The distribution volume with the porous versus the standard needle did not differ significantly between the 10 minute and the 100-120 minute infusion.

The porous needle was tested with injection on one side of the prostate and its performance compared with that of a standard needle injecting into the other side of prostate. The fraction of infused solution in the tissue was greater with the porous needle by almost 3-fold (*p* = 0.03) compared with standard needle. While the volume of distribution was greater with porous needle than the standard needle (80% higher), no statistically significant association could be determined with this small sample size. A Cohen’s *d* was calculated to be .6 (medium to large) and .79 (large) for porous needle vs standard needle for all grouped 1 and 2cm porous needles and 2cm porous needle, respectively, indicating that this finding will be significantly different if this trend continues. A sample size to achieve a power >.80 and alpha error < .05 is estimated to be a total of 35 for each group, or roughly an additional 20 samples to achieve a p value < 0.05. For the 2cm porous needle, a total of 26 samples in each group would be needed.

## Discussion

The purpose of a prostatic infusion is to deliver a planned dose to a planned targeted region. There are multiple pathways for flow of fluid carrying the agent that frustrate this goal, and therefore one needs a strategy to overcome these. Backflow does not seem to be an issue for the porous needle, and other strategies exist that can significantly limit backflow, though such approaches may create other procedural constraints. So, while backflow is an issue for standard needles at the high flow rates needed for clinical acceptability of the procedure, it does not appear a fundamental technological problem for intraprostatic infusions. However, there is a multiplicity of preferred pathways that lead to the urethra, as well as to the boundary of the prostate. Such ductal losses certainly suggest the benefit of multiport needle(s), so that one misplaced port does not vitiate the entire infusion, or multiple infusions with multiple needles, as is more customary. However, these ductal losses are not eliminated with such precautions and must be tolerated. The goal of the procedure is to ensure adequate dosing of the target while accepting therapeutic agent loss within and beyond the prostate. This has implications for the allowed toxicity of any potential agent similar to considerations for systemically administered agents. Outflow, in the sense of volume transmission of agents reaching the capsule and beyond, is by itself not a major issue if the backflow and ductal losses can be constrained. Obstacles are an issue: they will need to be identified in the imaging and multiple needle trajectories may become necessary to ensure distribution into nodules, requiring infusion with therapeutic agent.

There are several limitations of ex-vivo studies, such as the lack of tissue perfusion and any other in-vivo infusion distribution contributing factors. The number of prostates infused is small as mentioned; a larger sample may define greater difference between the porous needle and the standard needle. Another key limitation is the impact of translation to clinical patient therapy delivery that will influence ability to place and stabilize the needles during infusion. Thus, while these ex-vivo results are instructive of prostatic injection distribution, clinical studies are required to obtain reliable representation as to the relative contribution of the different flow mechanisms identified in these ex-vivo intraprostatic injections. However, these ex-vivo and prior comparative results of porous and standard needles indicate the porous needles provide improved distribution with the prostate [9, 10], including one in-vivo study [11], all of which have shown porous needle superiority over standard needle injections.

The results also indicate a need for pre-operative imaging and planning for placement of needles. We can envisage a hierarchy of approaches for increasing complexity and precision. We can begin with the best pre-operative imaging to reveal the structures in the prostate that affect flow and offer guidelines for placement of needles, and of flow rates, that are likely to avoid failure of infusions. The surgeon then reviews this information when placing the needles. However, it has become increasingly acceptable clinically to offer MR-fused 3D ultrasound guidance in biopsies of the prostate. This same technology can be used for infusions, wherein a plan is created as an overlay and fused with the real time 3D ultrasound so that the surgeon has a plan as guidance or information directly in view.

## Conclusions

This study demonstrated that prostatic tissue is anatomically heterogenic, which presents considerable challenge to achieving a desired distribution of injected agents, particularly from a standard needle. The complexity of flow pathways suggests that preoperative imaging and pre-infusion treatment planning to manage injectate distribution heterogeneity for consistent therapeutic results will be of potential value. The porous needle permitted greater fractional distribution of an injected complex agent compared to the standard needle. This approach to intraparenchymal therapy of prostate disease appears to warrant further investigation in the in vivo setting.

## Additional files

Additional file 1: **Table S1a.** N1 Subject Specific Data. Represents subject-specific injection data detailing the porous needle with flow rates, backflow parameter with inclusion of fraction and volume distribution for each subject in the porous needle cohort (N1). Presence of leakage to anatomic site and anatomic variation is noted for the cohort. Note: No. 4 describes standard needle parameters. (DOCX 15 kb)

Additional file 2: **Table S1b.** N2 Subject Specific Data. Represents subject-specific injection data detailing the standard needle with flow rates, backflow parameter with inclusion of fraction and volume distribution for each subject in the standard needle cohort (N2). Presence of leakage to anatomic site and anatomic variation is noted for the cohort. Note: No. 10 and No. 11 describe porous needle parameters. (DOCX 16 kb)

## Abbreviation

Gad-HAS: Gadolinium chelate bound to human serum albumin
