# Supplementary material for: A pilot study in intraparenchymal therapy delivery in the prostate: a comparison of delivery with a porous needle vs standard needle
Source: BMC Urol. 2018 Jul 28;18:66. doi: 10.1186/s12894-018-0378-8 (PMC6064133; doi:10.1186/s12894-018-0378-8)
Supplement: Supplementary file 1 — Table S1a. N1 Subject Specific Data. Represents subject-specific injection data detailing the porous needle with flow rates, backflow parameter with inclusion of fraction and volume distribution for each subject in the porous needle cohort (N1). Presence of leakage to anatomic site and anatomic variation is noted for the cohort. Note: No. 4 describes standard needle parameters. (DOCX 15 kb) [file 12894_2018_378_MOESM1_ESM.docx]

| Table S1a. *N1 Subject Specific Data* | | | | | | | | | |
| --- | --- | --- | --- | --- | --- | --- | --- | --- | --- |
|  |  | N1 | | | | | | | |
| # | Date | Cath1 (cm) | Flow rate (μL/min) | Backflow (mm) | Fraction in tissue | Vd (μL) | Vd/Vi | Leakage to Urethra | Nodule |
| 1 | 10/13/13 | 2 | 150 | — | 0.35 | 2824 | 1.9 | Large | In |
| 2 | 1/6/14 | 2 | 150 | — | 0.28 | 2858 | 1.9 | Moderate | Adjacent |
| 3 | 1/8/14 | 2 | 150 | — | 0.15 | 1631 | 1.1 | NO |  |
| 4 | 2/24/14 | Needle,N2 | 50 | 9 | 0.07 | 220 | 0.4 | NO | Adjacent |
| 5 | 3/3/14 | 1 | 100 | — | 0.36 | 1760 | 1.8 | Moderate | Several |
| 6 | 3/31/14 | 1 | 100 | — | 0.08 | 666 | 0.7 | Minor | ↓ T2w |
| 7 | 6/9/14 | 2 | 100 | — | 0.29 | 1731 | 1.7 | NO |  |
| 8 | 7/9/14 | 1 | 10 | — | .021 | 519 | 0.5 | Large |  |
| 9 | 9/17/14 | 1 | 10 | — | 0.35 | 2342 | 2.0 | NO |  |
| 10 | 10/8/14 | 1 | 10 | — | .019 | 925 | 0.8 | Minor |  |
| 11 | 10/29/14 | 2 | 10 | — | 0.66 | 2938 | 2.4 | Minor | Adjacent |
| 12 | 12/15/14 | 2 | 100 | — | 0.05 | 251 | 0.3 | Moderate |  |
| 13 | 12/17/14 | 2 | 100 | — | 0.47 | 1837 | 1.8 | Moderate |  |
| 14 | 1/7/15 | 1 | 100 | — | 0.31 | 1407 | 1.4 | NO | Adjacent |
| 15 | 1/26/15 | 1 | 100 | — | 0.41 | 2156 | 2.2 | Moderate | Near |
| 16 | 4/6/15 | 1 | 100 | — | 0.01 | 158 | 0.2 | NO |  |

NO = not visible in the MR contrast imaging, i.e. negligible.
