# Supplementary material for: A pilot study in intraparenchymal therapy delivery in the prostate: a comparison of delivery with a porous needle vs standard needle
Source: BMC Urol. 2018 Jul 28;18:66. doi: 10.1186/s12894-018-0378-8 (PMC6064133; doi:10.1186/s12894-018-0378-8)
Supplement: Supplementary file 2 — Table S1b. N2 Subject Specific Data. Represents subject-specific injection data detailing the standard needle with flow rates, backflow parameter with inclusion of fraction and volume distribution for each subject in the standard needle cohort (N2). Presence of leakage to anatomic site and anatomic variation is noted for the cohort. Note: No. 10 and No. 11 describe porous needle parameters. (DOCX 16 kb) [file 12894_2018_378_MOESM2_ESM.docx]

| Table S1b. *N2 Subject Specific Data* | | | | | | | | | |
| --- | --- | --- | --- | --- | --- | --- | --- | --- | --- |
|  |  | N2 | | | | | | | |
| # | Date | Cath2 (cm) | Flow rate (μL/min) | Backflow (mm) | Fraction in tissue | Vd (μL) | Vd/Vi | Leakage to Urethra | Nodule |
| 1 | 10/13/13 | Needle | 150 | 5^a^ | 0.35 | 2824 | 1.9 | Large | In |
| 2 | 1/6/14 | Needle | 150 | 11 | 0.28 | 2858 | 1.9 | Moderate | Adjacent |
| 3 | 1/8/14 | Needle | 150 | 4 | 0.15 | 1631 | 1.1 | NO |  |
| 4 | 2/24/14 | Needle | 50 | 1^a^ | 0.07 | 220 | 0.4 | NO | Adjacent |
| 5 | 3/3/14 | Needle | 100 | 13^b^ | 0.36 | 1760 | 1.8 | Moderate | Several |
| 6 | 3/31/14 | Needle | 100 | 7^a^ | 0.08 | 666 | 0.7 | Minor | ↓ T2w |
| 7 | 6/9/14 | Needle | 100 | 7 | 0.29 | 1731 | 1.7 | NO |  |
| 8 | 7/9/14 | Needle | 10 | 4^a^ | .021 | 519 | 0.5 | Large |  |
| 9 | 9/17/14 | Needle | 10 | 3^a^ | 0.35 | 2342 | 2.0 | NO |  |
| 10 | 10/8/14 | 2,N1 | 10 | — | .019 | 925 | 0.8 | Minor |  |
| 11 | 10/29/14 | 2,N1 | 10 | — | 0.66 | 2938 | 2.4 | Minor | Adjacent |
| 12 | 12/15/14 | Needle | 3 Boluses | 17^b^ | 0.05 | 251 | 0.3 | Moderate |  |
| 13 | 12/17/14 | Needle | 3 Boluses | 3 | 0.47 | 1837 | 1.8 | Moderate |  |
| 14 | 1/7/15 | Needle | 3 Boluses | 2^a^ | 0.31 | 1407 | 1.4 | NO | Adjacent |
| 15 | 1/26/15 | Needle | 3 Boluses | 6,13^b^ | 0.41 | 2156 | 2.2 | Moderate | Near |
| 16 | 4/6/15 | Needle | 100 | 0 | 0.01 | 158 | 0.2 | NO |  |

^a^ backflow reaches proximal surface

^b^ backflow reaches a transverse leakage path

NO = not visible in the MR contrast imaging, i.e. negligible.
